# Destabilized calcium dynamics visualized using the genetically-coded probe GCaMPJ in intact hearts of Calstabin2-null mice

**DOI:** 10.3389/fphys.2026.1787427

**Published:** 2026-04-29

**Authors:** Yixuan Liu, Youkun Bi, Jun Wang, Qi Yuan, Yu Rao, Fengchao Wang, Shiqiang Wang, Guangju Ji

**Affiliations:** 1 Institute of Biomedical Research, Henan Academy of Sciences, Zhengzhou, China; 2 School of Life Sciences, Zhengzhou University, Zhengzhou, China; 3 Institute of Biophysics, Chinese Academy of Sciences, Beijing, China; 4 Department of Medical Genetics, Life Sciences Institute, University of British Columbia, Vancouver, BC, Canada; 5 Department of Physiology and Cellular Biophysics, Columbia University, New York, NY, United States; 6 Ministry of Education (MOE) Key Laboratory of Protein Sciences, School of Pharmaceutical Sciences, Tsinghua University, Beijing, China; 7 National Institute of Biological Sciences, Beijing, China; 8 State Key Laboratory of Membrane Biology, College of Life Sciences, Peking University, Beijing, China

**Keywords:** arrhythmias, Ca^2+^ indicators, Calstabin, GCaMPJ, RyR

## Abstract

Optical imaging of intracellular calcium ions (Ca^2+^) using Ca^2+^-sensitive dyes or genetically encoded Ca^2+^ indicators (GECIs) offers high spatial and temporal resolution, and has revealed several critical mechanisms of Ca^2+^ signaling in cardiomyocytes. However, little progress has been made in the imaging of global Ca^2+^ events in intact mammalian hearts. To investigate the precise mechanisms of whole-heart arrhythmogenesis, highly sensitive macroscopic imaging tools are strictly required. Here, we utilized transgenic mice with cardiac-specific expression of GCaMPJ, an optimized high-fidelity Ca^2+^ indicator without cardiotoxicity. This enhanced sensitivity enabled the macroscopic visualization of complex spiral and ripple Ca^2+^ waves in intact hearts. Importantly, by deploying this advanced imaging platform in combination with genetic knockout and targeted protein degradation, we established the critical biological role of Calstabin in modulating Ca^2+^ cycling in the intact heart. We further demonstrated that the pharmacological stabilization of the ryanodine receptor (RyR) complex prevents profound spatial discordance and ventricular arrhythmias involving chaotic Ca^2+^ events.

## 1 Introduction

Calcium (Ca^2+^) plays a fundamental role in cardiac excitation-contraction coupling (Bers, 2002; Eisner et al., 2017; Landstrom et al., 2017). Cardiac Ca^2+^ enables a transmembrane gradient distribution that dominates secretion (Mayourian et al., 2018), excitability (De Smet et al., 2021), and contraction (Eisner et al., 2017). Altered Ca^2+^ handling has been associated with several heart diseases including arrhythmias and heart failure (Dridi et al., 2020). Fluorescence-based intracellular Ca^2+^ imaging is the most commonly used method for monitoring Ca^2+^ concentrations in living cells and organs (Kaestner et al., 2014; Mehta and Zhang, 2015; Sparrow et al., 2019). Ca^2+^ imaging typically uses Ca^2+^-sensitive fluorescent dyes, including small organic molecules and genetically encoded Ca^2+^ indicators (GECIs) (Semyanov et al., 2020). GECIs offer numerous advantages over small organic dyes, such as integrated Ca^2+^ imaging at the tissue and organ level and efficient targeting of specific cell types or subcellular compartments. GECIs are composed of a Ca^2+^-binding domain and one or two fluorescent proteins (Tian et al., 2009; Entcheva and Kay, 2021). The pioneering development of the earliest genetically engineered Ca^2+^ probes relied on fluorescence resonance energy transfer (FRET) between two distinct fluorescent proteins to report intracellular Ca^2+^ fluctuations (Miyawaki et al., 1997; Wilms and Häusser, 2014). Subsequently, a major paradigm shift occurred with the engineering of single-fluorescent-protein GECIs, wherein Ca^2+^ binding-dependent changes directly modulate the fluorescence intensity of a circularly permuted fluorescent protein (cpFP) within the chromophore environment (Nagai et al., 2001). Building upon this foundational milestone, the single-fluorophore toolkit has undergone continuous technical refinement. Recent advancements have dramatically expanded these capabilities, yielding highly sensitive and fast-response indicators that enable the precise tracking of complex Ca^2+^ dynamics across various physiological systems (Zhang et al., 2023).

Among the GECIs, the GCaMP family has the broadest application and is used in different tissues and model organisms (Tallini et al., 2006; He et al., 2008; Chen et al., 2013b; Shang et al., 2014). GCaMP was constructed based on a central circularly permutated enhanced GFP (cpEGFP), with calmodulin (CaM) at its C-terminal and M13 peptide at its N terminal (Chen et al., 2013b; Yang et al., 2018). Since the advent of GCaMP1 in 2001 (Nakai et al., 2001), scientists have attempted to optimize the affinity, signal-to-noise ratio, and dynamics of GCaMP through structure-based mutations and neuro-based screening methods and have successively developed GCaMP2 to CaMP6 to meet Ca^2+^ imaging requirements in various scenarios (Chen et al., 2013b; Dana et al., 2019; Nasu et al., 2021). GCaMP is one popular type of genetically-encoded Ca^2+^ indicator, most owing to its capability of Ca^2+^ measurement with impressive signal-noise ratio (SNR) and rapid response kinetics after continuous improvements and updates from GCaMP, especially GCaMP6 (Yang et al., 2018). Because of this, GCaMP6 has dominated the application of GECIs for some time. However, GCaMP6 has a limited dynamic range, which can lead to signal saturation or loss of sensitivity in some experimental conditions, especially when facing stress challenges. It also faces challenges in monitoring Ca^2+^ transients and sparks in an intact heart (Chen et al., 2013a; Garcia et al., 2017). Optimization of the existing Ca^2+^ indicator is always a crucial issue. In our previous study, an optimized GECI named GCaMPJ was developed based on the GCaMP3 skeleton, realizing enhanced fluorescence intensity and Ca^2+^ affinity *in vivo* (Chen et al., 2013b). The crystal structure of GCaMPJ revealed that the single point mutation D380Y directly strengthens the hydrogen bond network, and GCaMPJ exists as a monomer after binding to Ca^2+^. Our previous research proposed an efficient tool for mapping Ca^2+^ signals in intact organs to further improve GCaMP sensors.

Altered Ca^2+^ handling is responsible for adverse events in the heart such as cardiac arrhythmias, atrioventricular block, myocardial infarction, and heart failure (Synetos et al., 2016; Landstrom et al., 2017). The ryanodine receptor (RyR) regulates Ca^2+^ activity and its dysfunction leads to ventricular arrhythmias (Yuan et al., 2016; Zhao et al., 2017). During pathological processes such as heart failure and arrhythmogenesis, chaotic Ca^2+^ waves have been well-monitored in cardiomyocytes. However, investigating global Ca^2+^ events in intact hearts with high spatiotemporal resolution remains challenging using conventional low-molecular-weight sensors. In addition, Calstabin specifically binds to and stabilizes cardiac RyRs, further preventing Ca^2+^ leakage events (Zhao et al., 2017). The Calstabin family contains Calstabin1 (also named FK506-binding protein 12 [FKBP12]) and Calstabin2 (also named FKBP12.6), which specifically bind to RyR1 and RyR2, respectively (Huang et al., 2006; Kang et al., 2008). It is well established that Calstabin stabilizes RyR2 function. The depletion or abnormal dissociation of Calstabin2 from the RyR2 macromolecular complex causes a prominent diastolic SR Ca^2+^ leak, which severely impairs intracellular Ca^2+^ cycling and directly triggers fatal ventricular arrhythmias and sudden cardiac death (Marx et al., 2000; Sood et al., 2008). Furthermore, the critical nature of the RyR2-Calstabin interaction has been recently validated at the human single-cell level. Studies utilizing CRISPR/Cas9 to induce point mutations in the RyR2 FKBP-binding site have demonstrated that such disruptions severely impair Ca^2+^ signaling, enhancing the development of aberrant Ca^2+^ releases and Ca^2+^ sparks in human induced pluripotent stem cell-derived cardiomyocytes (hiPSC-CMs) (Fernández-Morales et al., 2022). Because this depletion reliably reproduces the pathological substrate of arrhythmias, the Calstabin2 knockout mouse serves as an ideal and appropriate *in vivo* model for our study to investigate and visualize these chaotic Ca^2+^ events using high-resolution tools. However, despite these cellular insights, the function of Calstabin in modulating global Ca^2+^ cycling in the intact heart remains to be fully elucidated.

To elucidate the precise function of Calstabin in modulating global Ca^2+^ cycling in the intact heart, highly sensitive macroscopic imaging tools are required. In this study, we utilized transgenic mice with cardiac-specific expression of GCaMPJ, an optimized sensor that overcomes the dynamic range limitations of earlier variants. By deploying this high-fidelity tool, we successfully traced complex arrhythmogenic Ca^2+^ patterns (including macroscopic spiral and ripple waves) in intact hearts. Furthermore, using both Calstabin2 knockout models and PROTAC-mediated protein degradation (Sun et al., 2019; Zeng et al., 2021), we established the critical role of the Calstabin-RyR2 complex in maintaining Ca^2+^ homeostasis and preventing ventricular arrhythmias under stress conditions.

## 2 Methods

### 2.1 Generation of transgenic mice and breeding

ROSA26 refers to a safe anchorage that is widely used to achieve generalized expression in mice to avoid random insertion of expression elements (Wu et al., 2016). In addition, the α-myosin heavy chain (αMHC) promoter was selected to drive conditional expression of target genes in cardiomyocytes (Inagawa et al., 2012). Here, the ROSA26-αMHC fragment was ligated to GCaMPJ or GCaMP6s, and the product was placed upstream of the transcription terminator SV40 polyA (Knapp et al., 2019). The constructed transgene was purified and co-injected with the CRISPR/Cas9 system into mouse oocytes to achieve precise, site-specific targeted knock-in at the ROSA26 locus (Wu et al., 2016). This targeted approach ensures single-copy integration at the identical locus for both GCaMPJ and GCaMP6s, eliminating expression variations caused by random insertion sites. Animals were genotyped using primers for GCaMPJ or GCaMP6s and using western blotting. The Calstabin2 null mice utilized in this study are a globally established transgenic line. The generation and complete molecular validation of this knockout model, including Western blot confirmation of protein ablation, have been described previously (Wehrens et al., 2005). Standard PCR genotyping was routinely performed to confirm the homozygosity of the mice before crossing them with GCaMPJ mice (Wehrens et al., 2005). Additionally, Calstabin2 null mice were crossed with GCaMPJ mice to generate GCaMPJ/Calstabin2^-/-^ double-transgenic (DTG) mice. All mouse-relevant procedures were performed according to the Guide for the Care and Use of Laboratory Animals published by the U.S. National Institutes of Health (NIH Publication No. 85–23, revised 1996) (Swearengen, 2018), and approval was obtained from the Institute of Biophysics Committee for Animal Care (Approval No. SYXK2018028). All mice were housed under standard conditions (12 h light/dark cycle; humidity, 40%–60%; temperature, 22 °C) with ad libitum access to food and water.

### 2.2 Anesthesia and euthanasia

Adult mice weighing 25–30 g were utilized for all experiments. All mice were anesthetized before surgery with tribromoethanol (Sigma-Aldrich) through intraperitoneal injection at a dose of 20 mg/100 g (Roth et al., 2002). Under an appropriate degree of anesthesia, the mice were relaxed and showed no response to painful stimuli accompanied by normal respiratory and heart rates. Surgery was not performed before a satisfactory degree of anesthesia was achieved. Tribromoethanol was used to provide general anesthesia for non-terminal surgical procedures and echocardiography, as it maintains relatively stable hemodynamics. According to the euthanasia guidelines for small laboratory animals, CO^2^ narcosis was provided as rapid depression with analgesic and anesthetic effects on the mice (Shomer et al., 2020). Typically, the initial CO^2^ delivery to the microisolator was accomplished by opening the CO^2^ cylinder valve so that mice were slowly exposed to increasing levels of CO^2^ (e.g., displacing approximately 10%–30% of the chamber volume per min). Furthermore, neonates (up to approximately 10 d old) are resistant to euthanasia using CO^2^ because of their resistance to hypoxia. Here, cervical dislocation was performed under CO^2^-induced narcosis to euthanize the neonates.

### 2.3 Ca^2+^-GCaMPJ-binding kinetics

The absorbance spectra of GCaMPJ and GCaMP6s were recorded using a spectrofluorometer (Applied PhotoPhysics, UK) as described in our previous study (Chen et al., 2013b). To investigate the monitoring performance of GCaMPJ and GCaMP6s on Ca^2+^ activity, their expression plasmids were transiently transfected into HeLa cells, respectively, followed by stimulation with 100 μM ATP-Na^+^. The fluorescence intensity was then recorded using a confocal microscope.

### 2.4 Isolation of primary cardiomyocytes and Ca^2+^ imaging

Heparin (5 IU/g body weight) was administered through intraperitoneal injection for 20 min to allow the heparin to circulate to the heart. Mice were anesthetized using intraperitoneal injection of 20% ethyl carbamate (2 mg ethyl carbamate/g body weight), followed by disinfection with 75% ethanol and immobilization. For the terminal heart excision procedure, ethyl carbamate (urethane) was used as the terminal anesthetic prior to primary cardiomyocyte isolation. This agent was selected for the tissue-harvesting step because it has historically been used in terminal cardiovascular experiments and was considered suitable for heart collection before cardiomyocyte isolation. Cardiomyocytes were isolated according to a previously reported protocol (Larcher et al., 2018). Well-growing cardiomyocytes were labeled with 10 μM Fluo-4 AM (Thermo, Cat^#^F14201), as per the manufacturer’s manual at room temperature and under dark conditions for 15 min. The signal cardiomyocytes were locked using a confocal microscope (Zeiss 880) to linearly screen for Ca^2+^ sparks at an excitation wavelength of 488 nm. During the imaging process, the cardiomyocytes were continuously perfused with an extracellular solution containing (in mM): 140 NaCl, 3 KCl, 2 CaCl2, 1 MgCl2, 10 HEPES, 10 glucose, 0.0005 TTX, 1 4-AP, and 0.005 glybenclamide (pH 7.4 adjusted with NaOH). The collected data were subjected to SparkMater in ImageJ software for parametric analysis. The cardiomyocytes of GCaMPJ or GCaMP6s mice were subjected to all operations without Fluo-4 AM staining to record Ca^2+^ sparks.

### 2.5 Echocardiography analysis

ECHO analysis was performed using the Vevo 770 (VisualSonics, Canada) and a matching probe (30 MHz). Briefly, mice were anesthetized with tribromoethanol and fixed at 37 °C constant temperature platform. The probe was placed on the left side of the sternum to image the long axis of the left ventricle, and a 90° rotation of the probe was used to image the short axis of the left ventricle. All data were visualized using the M model of the Vevo 770. The built-in software, Vevo 770, was used to quantify the measured parameters.

### 2.6 Langendorff perfusion and global ischemia–reperfusion protocol

Following anesthesia and heart excision, the aorta was rapidly cannulated and the heart was mounted on a Langendorff perfusion system. Hearts were retrogradely perfused at a constant pressure of 80 mmHg with modified Krebs–Henseleit buffer (KHB). The perfusate was continuously gassed with 95% O_2_/5% CO_2_ to maintain pH 7.4, and temperature was maintained at 37 °C using a water-jacketed heating system. After a 20-min stabilization period, global no-flow ischemia was induced by completely stopping coronary perfusion for 30 min. Throughout ischemia, hearts remained immersed in the warmed buffer bath to minimize cooling. Reperfusion was initiated by restoring KHB flow. For pharmacological intervention, S107 (10 μM) or vehicle was administered via the perfusate at the onset of reperfusion and maintained throughout the reperfusion phase. Optical mapping and ECG were acquired continuously, with analyses focused on arrhythmogenic Ca²^+^ activity occurring during the first 2 min of early reperfusion.

### 2.7 Optical mapping of the heart

Previous studies have documented a protocol for Ca^2+^ imaging in an intact heart. To avoid the potential adverse effects of pharmacological uncouplers on physiological excitation-contraction coupling, motion artifacts during macroscopic optical mapping were suppressed via mechanical stabilization. Briefly, the hearts were retrogradely perfused and suspended in a custom-designed fluid-tight optical chamber equipped with a front glass window. The distance between the aortic cannula and the glass window was accurately adjusted so that the ventricular epicardium was gently but firmly pressed against the glass. This physical immobilization effectively restricted myocardial movement and suppressed motion artifacts while maintaining normal perfusion pressure to avoid ischemia. A physiological recorder described the real-time ECG by receiving signals generated by the positive and negative electrodes fixed in the right atrium and left ventricle, respectively (Baker et al., 2000; Tallini et al., 2006). Both GCaMPJ and GCaMP6s were excited at 488 nm. Macroscopic Ca^2+^ signals and complex wave patterns were captured using a SteREO Discovery V20 microscope (Zeiss) equipped with a high-speed CMOS camera (ORCA-Flash 4.0 LT) at an acquisition rate of 100 frames per second (fps), as well as a high-speed CCD (ANDOR iXON Ultra EMCCD) operating at the maximal frame rate. Ca^2+^ activity during arrhythmia was triggered by continuous pacing at 10 Hz. As soon as pacing-induced chaotic Ca^2+^ events were detected, 100 μL S107 (10 μM) (a drug that specifically reduces RyR channel Ca^2+^ leak) (Synetos et al., 2016; Kushnir et al., 2018) was perfused into the heart to observe the changes in Ca^2+^ activity. All operations were accompanied by ECG recordings.

### 2.8 *In vivo* PROTAC (RC32) administration

To acutely degrade Calstabin proteins *in vivo*, the specific PROTAC molecule RC32 was utilized. Based on established protocols (Sun et al., 2019), adult GCaMPJ transgenic mice were administered RC32 via intraperitoneal injection at a dose of 30 mg/kg (dissolved in 0.9% NaCl containing 5% DMSO and 10% castor oil) twice daily for one week. Control mice received an equivalent volume of the vehicle solvent.

### 2.9 Western blot

Mouse tissues were homogenized and lysed in cold RIPA buffer containing 1mM PMSF and protease inhibitor cocktail (Cat #5871; Cell Signaling Technology) for 30 min. The supernatant was collected after centrifugation at 12,000 rpm and 4 °C for 20 min. Total protein concentration was determined using the Bradford method (Sigma, Cat^#^B6916). The prepared protein and 5× loading buffer mixture was boiled for 15 min. All protein samples were homogenized and separated using sodium dodecyl sulfate-polyacrylamide gel electrophoresis (SDS-PAGE), transferred onto nitrocellulose membranes, and incubated with primary antibodies against GFP (to specifically detect the GCaMP sensor; Abcam, Cat# ab290) or GAPDH (as an internal loading control; Cell Signaling Technology, Cat# 5174). All primary antibodies were applied at a standard 1:1000 dilution. The membranes were then incubated with the appropriate horseradish peroxidase (HRP)-conjugated secondary antibodies (1:10,000, ZSGB-BIO, China) and the signals were detected using the SuperLumina ECL HRP Substrate Kit (Abbkine, Cat^#^ K22030). β-Actin or GAPDH was used as an internal reference to assess protein levels. Three independent trials were performed.

### 2.10 Statistical analysis

Statistical analyses were performed using GraphPad Prism software. The biological unit for each experiment (number of independent animals/hearts, N, versus number of cells, n) is explicitly defined in the respective figure legends. The results were analyzed using an unpaired Student’s t-test for comparisons between two groups, or a Tukey one-way ANOVA and two-way ANOVA for multiple comparisons. Data are expressed as the mean ± SEM. The *p-*values considered statistically significant were *p < 0.05, **p < 0.01, and ***p < 0.001.

## 3 Results

### 3.1 Characteristics of GCaMPJ *in vitro* and *in vivo*

**Figure 1:**
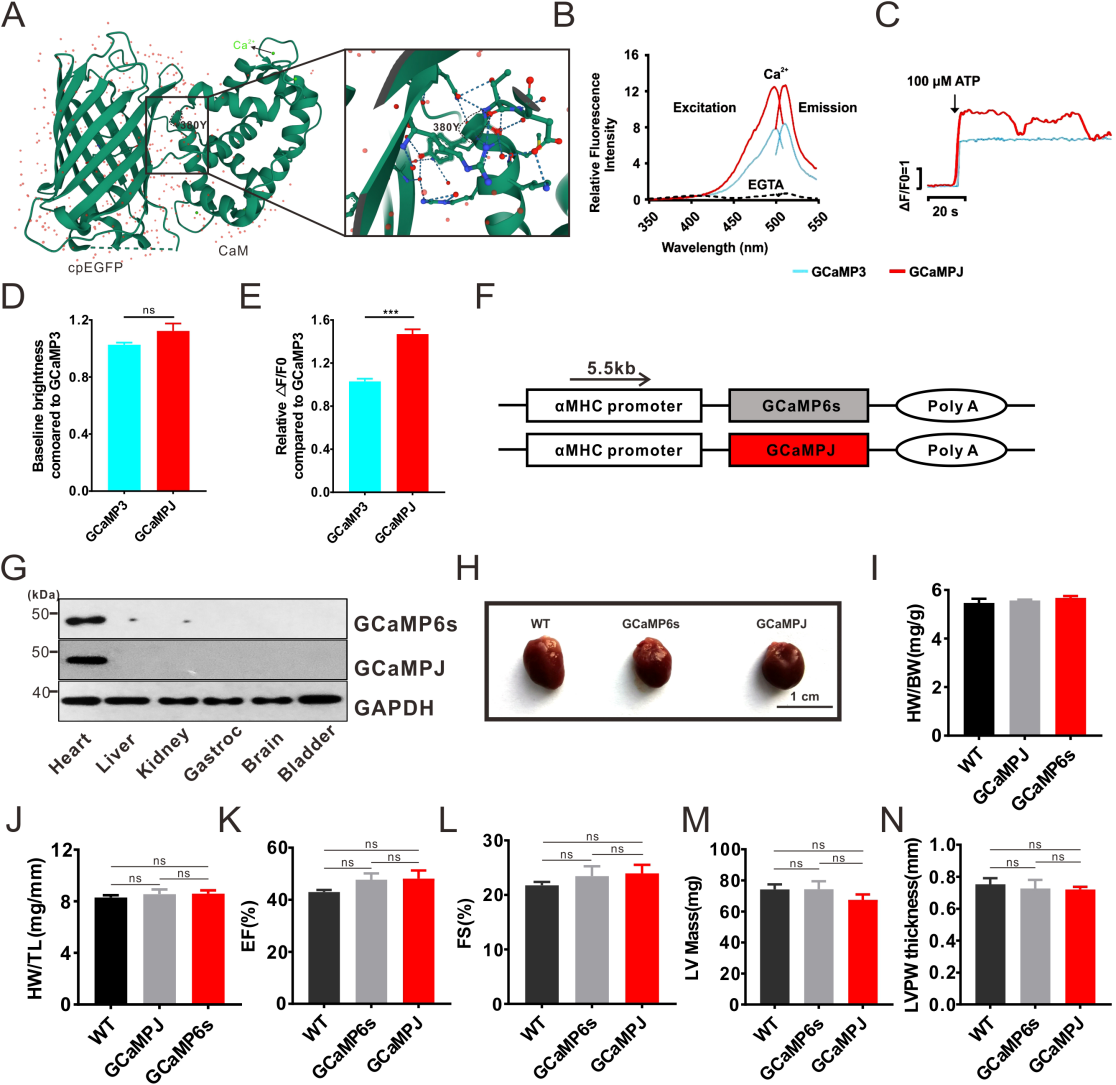
Characteristics of GCaMPJ *in vitro* and *in vivo.* **(A)** Crystal structure of GCaMPJ. Y, tyrosine. **(B)** Excitation and emission spectra of GCaMPJ and GCaMP3 in Ca^2+^-chelated (10 mM EGTA) and saturated (10 mM CaCl_2_) solution. **(C)** Relative fluorescence intensity changes in Hela cells expressing GCaMPJ and GCaMp3 when stimulated with 100 μM ATP. **(D)** Baseline fluorescence intensity (F0) was measured using FACS assay in Hela cells expressing GCaMPJ and GCaMp3. (n=70–100 from 3 independent transfection experiments for each group, p>0.05) **(E)**. Quantitative analysis of relative fluorescence intensity (n=70–100 from 3 independent transfection experiments for each group, p<0.001) **(F)** Schematic diagram of the construction of transgenic mice. **(G)** Expression detection of exogenous genes. **(H)** Morphologic observation of heart. **(I, J)** Quantitative analysis of heart weight/body weight (HW/BW) and heart weight/tibia length ratio (HW/TL). N = 8. **(K–N)** Echocardiography depicted the ejection fraction (EF), fractional shortening (FS), LV mass, and LVPW thickness of hearts in transgenic and normal mice. N = 8 independent mice per group. The data of **(D, E)** were expressed as mean ± SEM and subjected to two-tailed unpaired Student’s *t*-test. The data of **(I–N)** were analyzed using a Tukey one-way ANOVA. ^***^p<0.001.

Our previous research showed that GCaMPJ has a higher dynamic range and increased affinity for Ca^2+^ in GCaMP3 than in MOPS buffer (Chen et al., 2013b). The crystal structure of GCaMPJ was visualized and the position of tyrosine was specifically highlighted (Chen et al., 2013b) (**Figure 1A**). Purified GCaMPJ displayed similar fluorescence spectra to GCaMP3 at pH7.3 solution. There was an increase in the fluorescence signal from 0 to saturating Ca^2+^ between GCaMPJ (9-fold) and GCaMP3 (6.25-fold) (**Figure 1B**). Fluorescence activated cell sorting (FACS) analysis indicated that the baseline fluorescence of GCaMPJ was similar to that of GCaMP3 in HeLa cells (**Figures 1C, D**). However, GCaMPJ expression induced a 1.48-fold Ca^2+^ transient compared to GCaMP3 when stimulated with 100 μM adenosine triphosphate (ATP), resulting in Ca^2+^ release from the endoplasmic reticulum (ER) (**Figure 1E**). Thus, GCaMPJ exhibited a better performance in terms of Ca^2+^ affinity and fluorescence intensity *in vitro*.

The GCaMP6 series has broad applications in Ca^2+^ tracking in intact hearts (Yang et al., 2018). Among its variants, GCaMP6s is characterized by an optimal ΔF/F0. Here, GCaMP6s was selected as a comparison to evaluate the ability of GCaMPJ to track Ca^2+^ events in intact beating hearts. The mice specifically expressing GCaMPJ or GCaMP6s in cardiomyocytes were generated as shown in the description in **Figure 1F**. To realize the strengthened expression of GCaMPJ or GCaMP6s in hearts, a particular αMHC promoter with a strong and homogenous transgenic expression in all areas of the heart was chosen (Inagawa et al., 2012). The promoter was placed upstream of the inserted exogenous gene. Western blotting confirmed that the inserted genes were restricted to the myocardial tissue and were absent in other tissues (**Figure 1G**). Previous studies reported that part of GCaMP family had cardiotoxic resulting in significant cardiomegaly, like GCaMP2 (Tallini et al., 2006), although it can be used to monitor the Ca^2+^ activities in intact heart. Therefore, the morphology and physiological functions of the hearts of transgenic mice were investigated in our study. There was no cardiac hypertrophy compared to normal mice according to morphological observations (**Figure 1H**), heart weight/body weight (HW/BW) (**Figure 1I**), and heart weight/tibia length ratio (HW/TL) (**Figure 1J**). In addition, echocardiography revealed no significant differences in ejection fraction (EF), fractional shortening (FS), LV mass, and LVPW thickness between transgenic mice and normal mice (**Figures 1K–N**).

### 3.2 GCaMPJ provides distinct advantages for Ca^2+^ monitoring in primary cardiomyocytes

**Figure 2:**
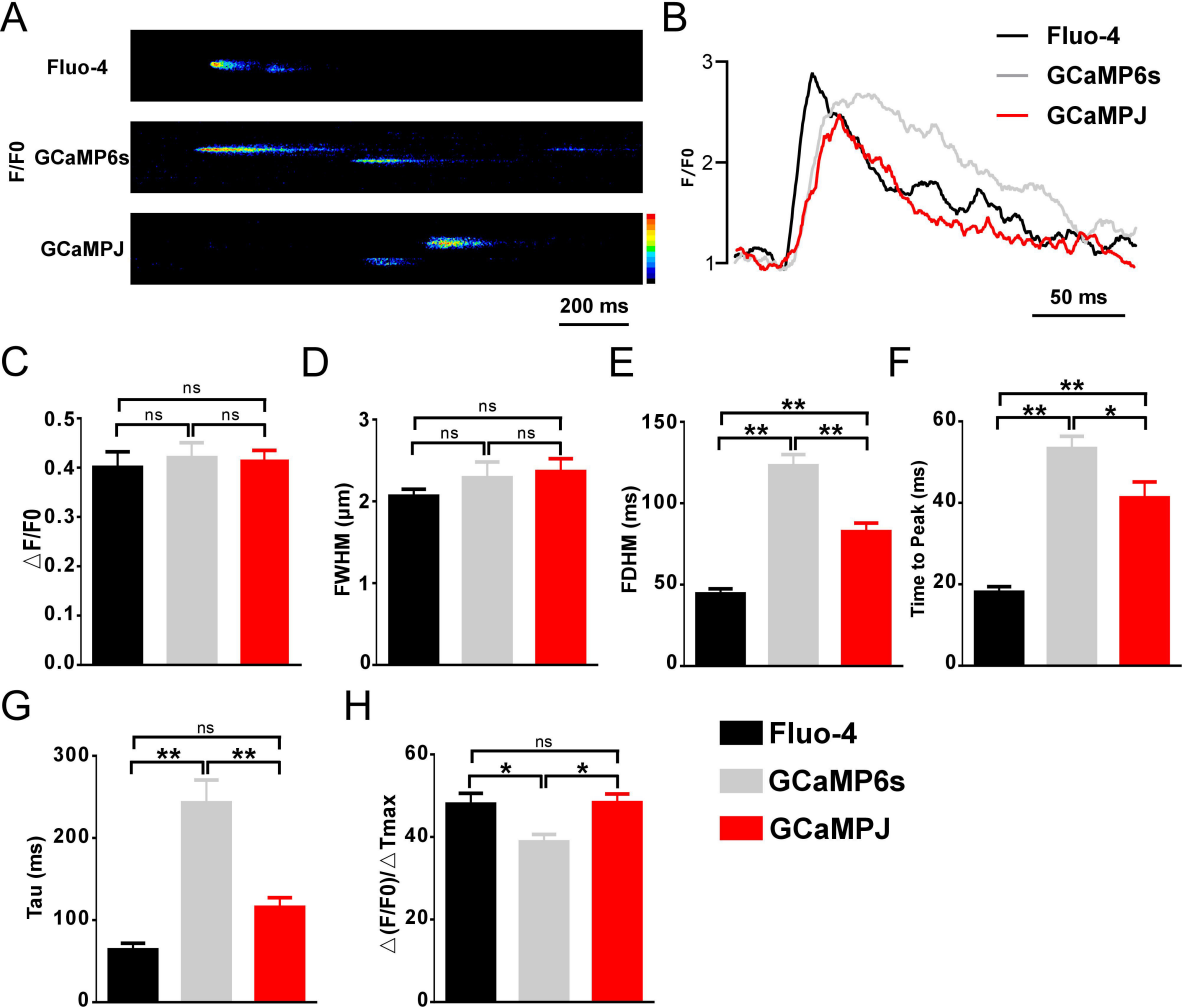
GCaMPJ provides distinct advantages for Ca^2+^ monitoring. **(A)** Representative micrographs of Ca^2+^ sparks. **(B)** Quantification of Ca^2+^ spark fluorescence intensity (F/F0). Here, F0 is the background baseline intensity measured in the recording solution without Ca^2+^ within 1 min, and F represents the actual fluorescence intensity. **(C)** The physiological amplitude of fluorescence intensity (△F/F0). Here, F0 represents the resting diastolic fluorescence of the cardiomyocyte. **(D)** Quantification of full width at half maximum (FWHM). **(E)** Quantitative assay of Full duration half maximum (FDHM). **(F)** The rise time of fluorescence intensity. **(G)** The half decay time of fluorescence intensity. **(H)** Speed change of fluorescence intensity. All statistical data were analyzed using a Tukey one-way ANOVA. Data were obtained from n = 10 isolated cardiomyocytes across N = 3 independent mice per group. Error bars represent the mean ± SEM. *p < 0.05, **p < 0.01.

The isolated primary cardiomyocytes from transgenic mice were subjected to confocal imaging to observe the properties of the Ca^2+^ sparks. Fluo-4 staining was used to characterize the Ca^2+^ signals. The results revealed that the Ca^2+^ spark intensity changes (F/F0) and full width at half maximum (FWHM) in transgenic mice were not different from those of Fluo-4 staining (**Figures 2A–D**). Furthermore, both GCaMPJ and GCaMP6s exhibited a delayed trend. However, GCaMP6s was far inferior to GCaMPJ in terms of time parameters. Significantly, the full duration half maximum (FDHM) was extended by 49.6% (**Figure 2E**), the time to reach the fluorescence peak was 23.37% faster (**Figure 2F**), and the half decay time was reduced by over 50% (**Figure 2G**). The rate of change in fluorescence intensity increased by 20.3% (**Figure 2H**). Spectroscopic studies showed that GCaMPJ had an elevated value compared to GCaMP6s in terms of (F-Fmin)/Fmin (ΔF/F0) (**Supplementary Figure 1A**), and similar situation occurs in the evaluation of Ca^2+^ affinity (**Supplementary Figure 1B**). In the case of recording spark frequency, both GCaMPJ and GCaMP6s had similar ability with Fluo-4 (**Supplementary Figure 2**). Overall, the monitoring data of Ca^2+^ sparks at the single cell level revealed that GCaMPJ was superior to GCaMP6s in terms of Ca^2+^ activity (**Figure 2**).

### 3.3 GCaMPJ enables highly sensitive Ca^2+^ imaging in intact hearts

**Figure 3:**
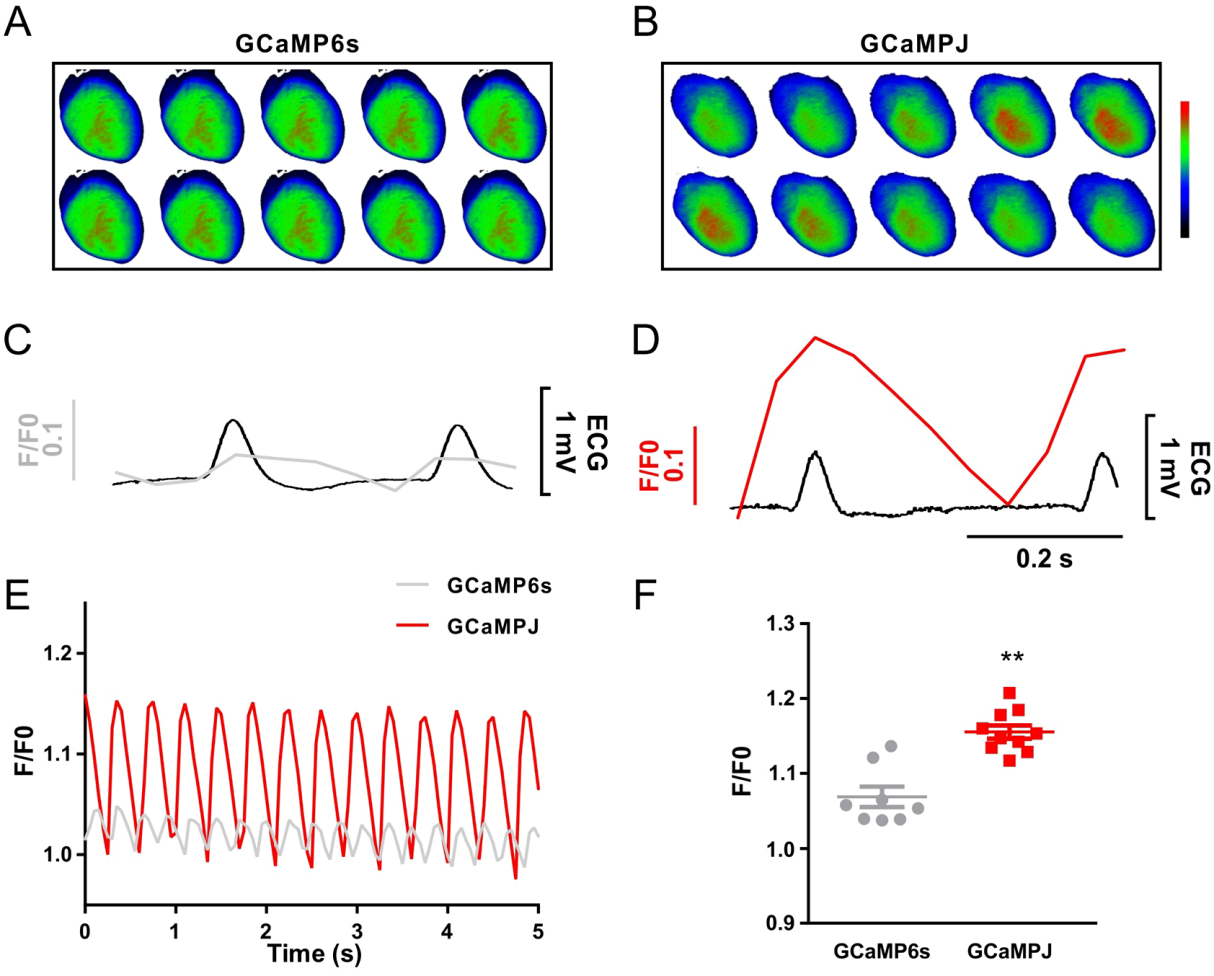
GCaMPJ enables highly sensitive Ca^2+^ imaging in intact hearts. **(A, B)** Representative photographs of Ca^2+^ signal changes on the surface of the heart during a cardiac cycle. **(C, D)** Ca^2+^ signal changes on the surface of the heart and ECG during a cardiac cycle corresponded to **(A, B)**, respectively. The horizontal time scale for the highly magnified single cardiac cycle traces in both **(C, D)** is 0.2 (s) In **(E)**, the x-axis label (‘Second’) indicates Time (s). The vertical scale bar in **(C, D)** represents a relative amplitude increment of 0.1, which aligns quantitatively with the absolute F/F0 peaks shown in the continuous 5 s recording **(E)** and the statistical summary **(F)**. **(E)** Comparison of Ca^2+^ signal changes on the surface of the heart within 5 (s) **(F)** Statistical analysis of fluorescence intensity. The number of GCaMPJ and GCaMP6s mice is 12 and 9, respectively. All statistical data were represented as mean ± SEM and performed two-tailed unpaired Student’s *t*-test. ^**^p<0.01.

While GECIs offer significant advantages for tissue-level imaging, some earlier GCaMP variants exhibited kinetic limitations compared to traditional small-molecule dyes such as Fluo-4 at the single-cell level (Tallini et al., 2006; Wu et al., 2019). However, Fluo-4 is difficult to permeate into dense tissues uniformly and fails to achieve macroscopic Ca^2+^ imaging in whole organs. Here, the Ca^2+^ imaging capability in the hearts of transgenic mice was investigated. The aortic reverse perfusion technique was used to isolate the normal beating heart. The surface Ca^2+^ fluorescence intensity was tracked using an ultrahigh-speed camera. The results indicated that GCaMPJ showed higher sensitivity to Ca^2+^ change than GCaMP6s in the intact heart (**Figures 3A, B**). GCaMPJ presented robust and steady Ca^2+^ signal fluctuations corresponding to the cardiac cycle, whereas the signal amplitude was profoundly attenuated and less distinct in GCaMP6s mice (**Figures 3C, D**). In addition, GCaMPJ showed an enhanced fluorescence signal intensity of Ca^2+^ changes compared to GCaMP6s on the heart surface within 5 s (**Figures 3E, F**). Therefore, GCaMPJ represents a highly effective alternative to GCaMP6s for macroscopic Ca^2+^ imaging in intact hearts. Having established this robust and sensitive imaging platform, we next utilized these transgenic mice to investigate the potential biological role of Calstabin in arrhythmic Ca^2+^ dynamics.

### 3.4 Stabilization of RyR2 prevents pacing-induced chaotic Ca^2+^ events in intact hearts

**Figure 4:**
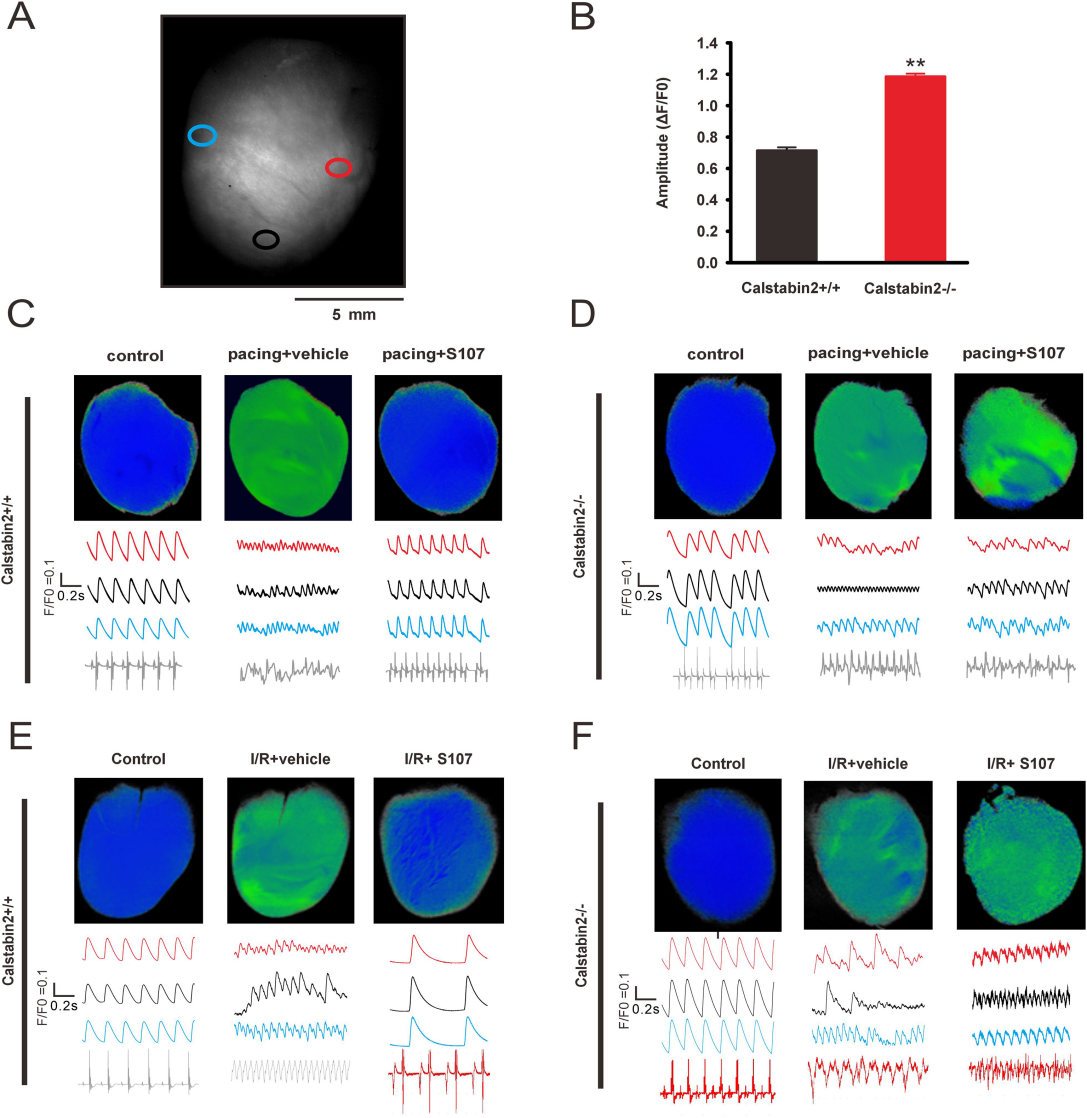
Stabilization of RyR2 prevents chaotic Ca^2+^ events in intact hearts. **(A)** Schematic diagram of the mouse heart. Circles represent the random detection area of the Ca^2+^ signal. **(B)** Amplitude comparison of Ca^2+^ activity on the surface of normal beating hearts from GCaMPJ/Calstabin2^+/+^ and GCaMPJ/Calstabin2^-/-^ mice. N = 5 independent hearts per group and all statistical data are represented as mean ± SEM using two-tailed unpaired Student’s t-test. ^**^p<0.01. **(C, D)** The cardiac Ca^2+^ activities of GCaMPJ/Calstabin2^+/+^ and GCaMPJ/Calstabin2^-/-^ mice under pacing stimulation and S107 intervention. **(E, F)** The cardiac Ca^2+^ activities of GCaMPJ/Calstabin2^+/+^ and GCaMPJ/Calstabin2^-/-^ mice with I/R and S107 intervention. **(C–F)** At the top is a schematic diagram of the overall cardiac Ca^2+^ activities. The red, black, and blue curves below represent the fluorescence intensity changes of three random areas described in **(A)**. The bottom trace shows the ECG, which was recorded simultaneously with the Ca^2+^ activities. The horizontal scale bar for the traces in **(C–F)** represents 0.2 (s) The baseline control hearts exhibited a spontaneous heart rate of approximately 300 bpm (5 beats per second). For pacing stimulation, hearts were subjected to burst pacing at a frequency of 10 Hz. For S107 rescue experiments in both pacing and I/R models, group-level incidence of chaotic Ca^2+^ events was quantified across N = 5 independent hearts per experimental condition.

Next, Ca^2+^ events in the ventricular arrhythmia heart induced by burst pacing was investigated, and an ECG was used to record a disordered heart rate (**Supplementary Figure 3**). Ca^2+^ re-entrant patterns were detected in three randomly selected regions of the heart, and chaotic Ca^2+^ events in intact hearts were recorded during arrhythmias (**Figure 4A**). In this study, ‘chaotic Ca²^+^ events’ refers to spatiotemporally disorganized Ca²^+^ activity observed during arrhythmias, characterized by loss of regular beat-to-beat periodicity and by regional heterogeneity in Ca²^+^ dynamics (e.g., intermittent spontaneous transients, oscillations, and/or alternans occurring in different regions of the same heart) (Song et al., 2015; Uzelac et al., 2017). This terminology is used descriptively and does not imply a formal demonstration of deterministic chaos. Rapid pacing is responsible for diastolic SR Ca^2+^ leakage when arrhythmia occurs, and RyR2 stabilization can correct these adverse events (Guo et al., 2012; Sun et al., 2019). As previously reported (Zhao et al., 2017), Calstabin2 stabilizes the Ca^2+^ release channel by binding to the RyR2 complex. Accordingly, the small molecule S107 (a specific Rycal) was utilized. Mechanistically, S107 does not directly block the RyR2 channel pore; rather, it specifically binds to the RyR2 complex to increase its binding affinity for Calstabin2, thereby inhibiting Calstabin2 dissociation and sealing the SR Ca^2+^ leak (Andersson et al., 2011). Crucially, because its mechanism relies entirely on the RyR2-Calstabin2 interaction, S107 requires the physical presence of Calstabin2 to exert its stabilizing effect. To validate this mechanism and determine the function of Calstabin2 underlying global chaotic Ca2+ events, Calstabin2^-/-^ mice were crossed with GCaMPJ mice to generate GCaMPJ/Calstabin2^-/-^ DTG mice. Calstabin2 depletion directly resulted in chaotic Ca^2+^ activity in the hearts of DTG mice, and significantly higher Ca^2+^ transient amplitude (**Figures 4B–D**) under pacing stress. As soon as pacing-induced global chaotic Ca^2+^ events were detected, the heart was perfused with 10 µM S107. Adverse Ca^2+^ events were abolished by S107 in the hearts of GCaMPJ/Calstabin2^+/+^ mice within 10 min. Group-level quantification showed that the incidence of pacing-induced chaotic Ca^2+^ events was reduced from 5/5 (vehicle) to 1/5 following S107 treatment (indicating successful prevention in 4 of 5 hearts). In contrast, chaotic Ca^2+^ events persisted in the hearts of GCaMPJ/Calstabin2-/- mice despite S107 intervention (incidence 5/5) (**Figures 4C, D**).

### 3.5 Stabilization of RyR2 prevents ischemia/reperfusion(I/R)-induced chaotic Ca^2+^ events in intact hearts

Reperfusion injury after ischemia results in ventricular arrhythmia (Wu et al., 2016). This pathological process is triggered by abnormal Ca^2+^ activity involving spontaneous Ca^2+^ release from RyR2, further activating the Na^+^/Ca^2+^ exchanger (NCX), which leads to arrhythmogenic delayed after-depolarization (DAD) (Blayney and Lai, 2009). Here, a documented protocol was used to achieve global ischemia of the heart for 30 min to investigate Ca^2+^ signals during arrhythmias that occurred in the first 2 min of reperfusion (Blayney and Lai, 2009). Simultaneous Ca^2+^ imaging and ECG records revealed chaotic Ca^2+^ activity and typical patterns of ventricular tachycardia, respectively. During the early reperfusion period, simultaneous multiparametric recordings revealed highly discordant regional behaviors. For instance, as representatively shown in **Figure 4E** (I/R+vehicle), some ventricular regions displayed pronounced beat-to-beat Ca^2+^ alternans (blue trace), while adjacent areas simultaneously exhibited either uncoordinated Ca^2+^ oscillations (black trace) or micro-alternans patterns of a much smaller magnitude (red trace). This profound spatial discordance and regional heterogeneity in Ca^2+^ signaling directly contribute to the arrhythmogenic substrate. These findings suggest that different patterns of Ca^2+^ signaling in different parts of the ventricle contribute to ventricular arrhythmias.

The effects of S107 on Ca^2+^ handling and ventricular arrhythmias following I/R in intact hearts were investigated, and the I/R procedure was followed as previous report (Wang et al., 2010). S107 intervention significantly increased Ca^2+^ transient amplitude and decreased the frequency of spontaneous Ca^2+^ transients compared to the vehicle, and the hearts displayed overall normal Ca^2+^ transients (Andersson et al., 2011). Quantitative analysis revealed that while the incidence of chaotic Ca^2+^ events was 5/5 in vehicle-treated hearts during reperfusion, there were no episodes of ventricular tachycardia or chaotic Ca^2+^ events during reperfusion with 10 µM S107 in GCaMPJ/Calstabin2+/+ mice (incidence 0/5; successful prevention in 5 of 5 hearts). However, S107 did not prevent abnormal Ca^2+^ events or ventricular tachycardia in the hearts of GCaMPJ/Calstabin2-/- mice (incidence 5/5) (**Figure 4F**). Collectively, these data indicate that RyR2-related SR Ca^2+^ leakage is crucial for ventricular arrhythmias during reperfusion, and Calstabin2 plays an essential role in regulating Ca^2+^ release in the heart.

### 3.6 Ca^2+^ dynamics monitoring in the ischemic heart

**Figure 5:**
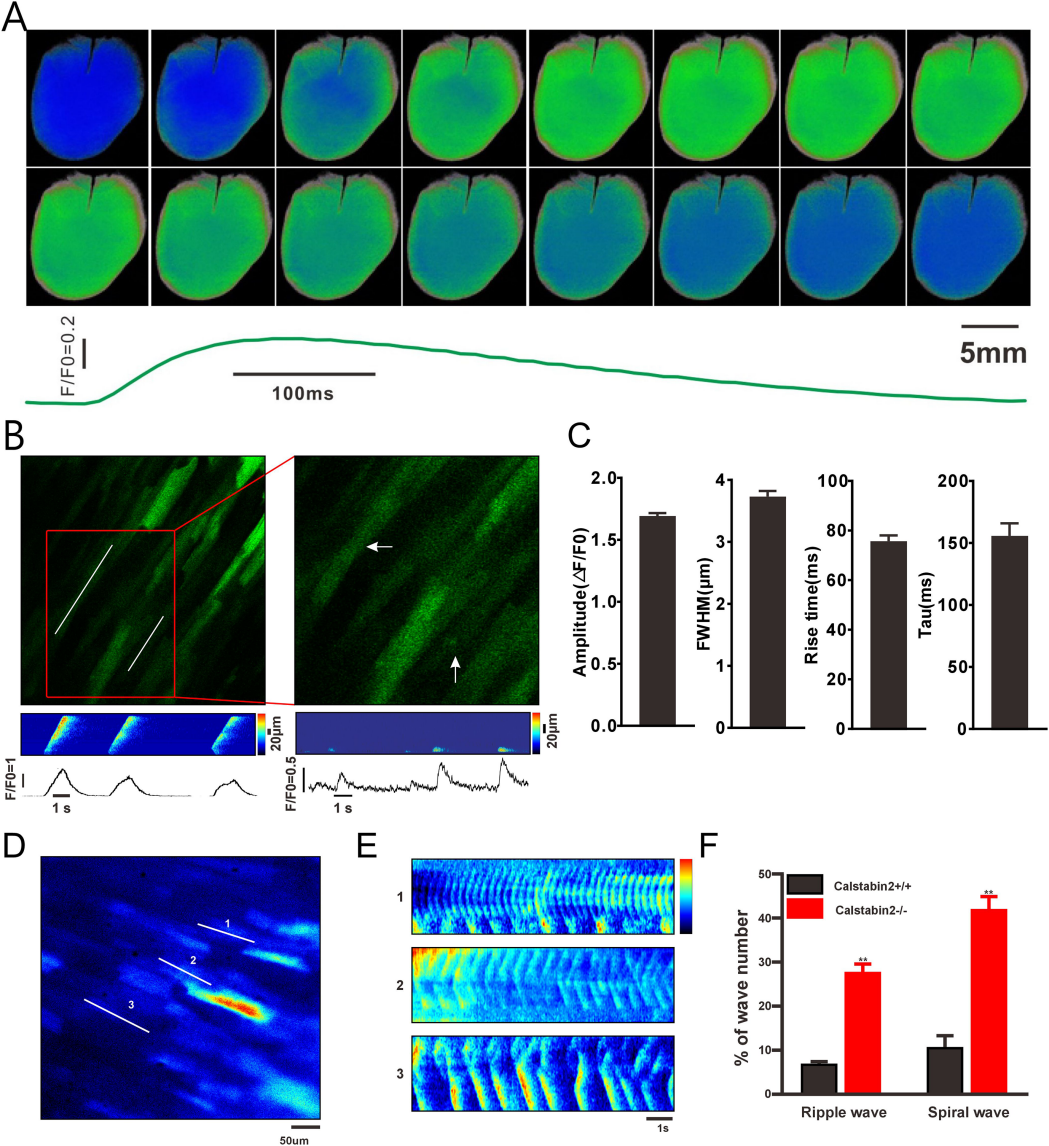
Ca^2+^ dynamics monitoring in ischemic heart. **(A)** Representative sequential photographs of the Ca^2+^ activities in intact hearts from αMHC-GCaMPJ mice during a cardiac cycle. **(B)** Highly localized Ca^2+^ transients and spark-like events were observed in the intact GCaMPJ hearts, as indicated by arrows. The lower panels show pseudo linescan images from where indicated by lines in the upper panel. **(C)** The Ca^2+^ spark parameters of the intact heart of αMHC-GCaMPJ mice. The amplitude was (1.71 ± 0.02) (ΔF/F0), full width at half maximum was (3.73 ± 0.17), rise time (taken from the point of rising to peak ΔF/F0) was (76.41 ± 7.52) ms, and the half time decay (tau) was (156.4 ± 18.58) ms. Data were collected from N = 5 independent hearts, with an estimated 30–50 localized Ca^2+^ events recorded per heart. Statistical parameters were calculated based on the independent biological replicates (N = 5) rather than pooled individual events. Data are expressed as mean ± SEM. **(D)** The highly localized Ca^2+^ release events induced by cardiac I/R in intact hearts of GCaMPJ/Calstabin2-/- mice. [1], [2], and [3] represent the Ca^2+^ activities of three distinct micro-domains, respectively. All data were subjected to a Tukey one-way ANOVA test. **(E)** The pseudo linescan (x–t) images from **(D)**. 1 represents Ca^2+^ripple waves, 2 and 3 represent spiral waves. **(F)** The incidence rate of ripple and spiral waves in GCaMPJ/Calstabin2^+/+^ and GCaMPJ/Calstabin2^-/-^ mice. All statistical data are represented as mean ± SEM and performed two-tail unpaired Student’s *t*-test. N = 8, ^**^p<0.01.

Transgenic mice expressing GCaMPJ in cardiomyocytes were selected to detect Ca^2+^ in intact hearts. A high-speed CMOS camera recorded global Ca^2+^ events in the left ventricular surface of a Langendorff-perfused heart. High signal-to-noise spontaneous Ca^2+^ events were recorded across multiple regions of the left ventricular surface of the TG mice (**Figure 5A**). Owing to the superior performance of GCaMPJ, highly localized Ca^2+^ transients and spark-like events were observed in micro-domains of intact hearts from GCaMPJ mice (**Figures 5B, C**, Video 1, and Video 2).

Intracellular Ca^2+^ signals were also captured using a high-speed CMOS camera at 100 fps in perfused intact hearts (**Figure 5D**, **Supplementary Video 3**). Two patterns of continuous Ca^2+^ dynamics were observed. One was a spiral wave, morphologically defined as a re-entrant rotor continuously rotating around a distinct unexcited core. The other pattern, termed a ripple wave, was defined as high-frequency, concentric oscillatory expansions originating from a focal ectopic pacemaker (**Figure 5E**). Although spiral and ripple waves have been previously reported, they are limited to detection at a highly localized level. Quantitative analysis revealed that Ca^2+^ ripple and spiral waves constituted approximately 28% and 42% of the total abnormal wave events, respectively, in GCaMPJ/Calstabin2^-/-^ mice, whereas these events were almost absent in GCaMPJ/Calstabin^2+/+^ mice during the early period of reperfusion (**Figure 5F**). Intriguingly, localized spiral and ripple waves were detected while a cardiac arrhythmia occurred. Because adjacent highly localized micro-domains exhibit different Ca^2+^ wave patterns, unsynchronized Ca^2+^ events are crucial for initiating and maintaining ventricular arrhythmias.

### 3.7 RC32 induced adverse Ca^2+^ events in GCaMPJ mice

**Figure 6:**
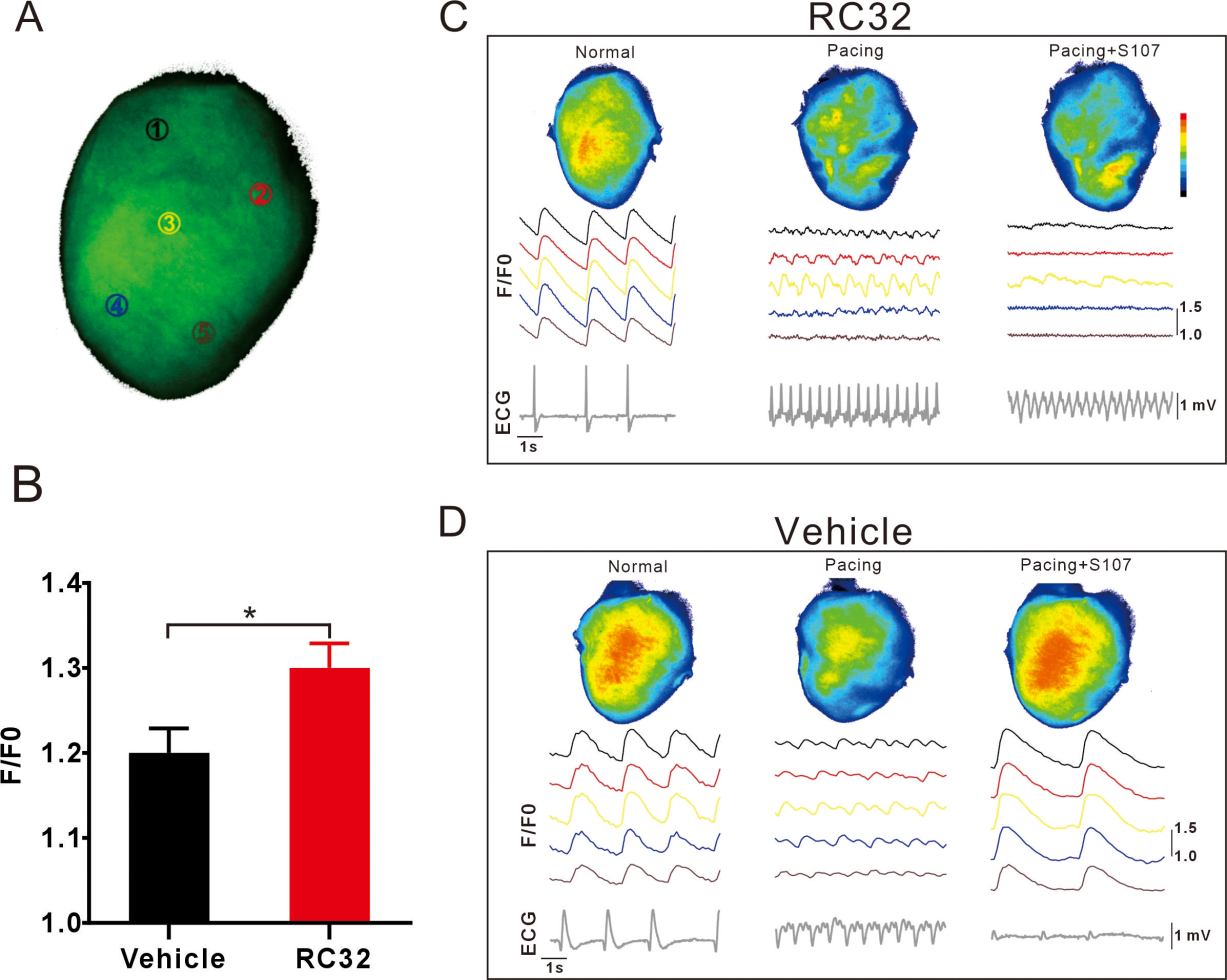
RC32 induced adverse Ca^2+^ events in GCaMPJ mice. **(A)** Schematic diagram of the mouse heart. Circles represent the random detection areas of the Ca^2+^ signal. **(B)** Ca^2+^-dependent fluorescence intensity on the surface of normal beating hearts from GCaMPJ and RC32-treated GCaMPJ mice. All statistical data are represented as mean ± SEM using two-tail unpaired Student’s *t*-test. N = 8, ^*^p<0.05. **(C, D)** The cardiac Ca^2+^ activities of RC32-treated and vehicle-treated GCaMPJ mice under pacing stimulation and S107 intervention. At the top is a schematic diagram of the overall cardiac Ca^2+^ activities, and the five curves below correspond to the fluorescence intensity changes of five areas described in **(A)**. The bottom trace shows the ECG, which was recorded simultaneously with the Ca^2+^ activities.

Our lab previously developed a small compound named RC32 that acutely degrades both Calstabin1 and Calstabin2 proteins using the PROTAC technique (Sun et al., 2019). To investigate the effect of RC32 on Ca^2+^ signals in intact hearts, GCaMPJ mice were administered RC32 for one week, as previously reported. The results revealed that the hearts of RC32-treated mice had higher Ca^2+^ fluorescence intensity than the control mice treated with the vehicle (**Figures 6A, B**). Pacing-induced arrhythmia was observed in the hearts of RC32-treated and vehicle-treated mice with chaotic Ca^2+^ events and abnormal ECG recordings (**Figures 6C, D**). As expected, S107 intervention rescued the disordered Ca^2+^ signals and restored heart rate to normal levels in the hearts of vehicle-treated mice. However, a similar situation was not observed in RC32-treated mice (**Figures 6C, D**). These data revealed that RC32 disrupted the Ca^2+^ steady state, interpreted as the perturbation of RyR2 caused by Calstabin degradation, which offers further the vital role of Calstabin in the heart.

## 4 Discussion

In this study, arrhythmia-related chaotic Ca^2+^ events in the intact hearts of GCaMPJ transgenic mice were recorded and it was suggested that stabilization of RyR2 contributes to the prevention of abnormal Ca^2+^ events. Previous studies have indicated that disturbances in intracellular Ca^2+^ levels give rise to ventricular arrhythmias (Blayney and Lai, 2009; Wang et al., 2010; Synetos et al., 2016; Dridi et al., 2020). This study extends the GECI-assisted visualization of intracellular Ca^2+^ signals to the intact heart at a highly localized micro-domain level. This provides new evidence that cardiac arrhythmias are closely correlated with the global chaotic Ca^2+^ events observed in intact hearts.

The most effective method for monitoring Ca^2+^ activity *in vivo* is a fluorescence-based method that typically uses Ca^2+^-sensitive small organic fluorescent molecules and GECIs (Tian et al., 2009; Dana et al., 2019). Compared with GECIs, small organic molecules have limitations in tracing Ca^2+^ signals in multicellular organs, such as complicated loading procedures and consumption of dye molecules (Wu et al., 2019). Therefore, GECIs are preferred for whole-heart Ca^2+^ imaging. GCaMPs are the most widely used GECIs and are being rapidly updated (Chen et al., 2013b). Numerous GCaMPs have been developed and applied in various laboratories. In 2006, GCaMP2 was the first to illustrate Ca^2+^ activity in the intact hearts of transgenic mice (Tallini et al., 2006). Since then, the optimization of GCaMPs has become an important research matter. Among the GCaMPs, the GCaMP6 series has superior Ca^2+^ indicator properties and broad applications (Garcia et al., 2017). While the GCaMP6 series (particularly variants such as GCaMP6f) features highly optimized dissociation rates and decay kinetics, GCaMPJ provides a distinct set of complementary advantages. Specifically, GCaMPJ demonstrates higher Ca^2+^ affinity and enhanced macroscopic fluorescence intensity. These properties, combined with the favorable kinetic parameters we observed compared to GCaMP6s at the single-cell level, facilitate the monitoring of small Ca^2+^ events in the dense tissue of intact hearts. In addition, there is no cardiotoxicity in GCaMPJ mice, and GCaMPJ expression does not require the induction of drugs and is therefore convenient in experimental settings compared with the previous version. Owing to its excellent properties, spiral and ripple localized spiral and ripple waves were captured in functioning hearts. Although Ewan et al (Fowler et al., 2018). described Ca^2+^ ripples in cardiomyocytes, they did not capture the ripple-shape as shown in our study.

Many studies have reported that chaotic intracellular Ca^2+^ activities give rise to cardiac arrhythmias (Blayney and Lai, 2009; Landstrom et al., 2017). Burst pacing (Fabritz et al., 2003) and I/R injury (Dobrev and Wehrens, 2018) are commonly used to induce cardiac arrhythmias in experimental animals. This pathological process is associated with the activity of the SR Ca^2+^ release channels, RyR2 (Huang et al., 2006; Blayney and Lai, 2009). Calstabin2, a regulatory protein that forms a complex with RyR2, modulates Ca^2+^ release by manipulating RyR2 ultrastructural organization (Huang et al., 2006; Kang et al., 2008). Previous studies have shown that S107, a RyR2-specific Rycal, prevents Calstabin2 depletion, thereby reducing Ca^2+^ leakage by stabilizing the RyR2-Calstabin2 interaction (Andersson et al., 2011; Fauconnier et al., 2011). RyR2 stabilization leads to the attenuation of Ca^2+^ leakage from the SR and prevents cardiac arrhythmias (Guo et al., 2012). Here, chaotic Ca^2+^ events in intact hearts during ventricular arrhythmias induced by pacing and I/R in cardiac GCaMPJ mice are described. S107 intervention eliminated chaotic Ca^2+^ activity and terminated ventricular arrhythmias. However, S107 was ineffective in GCaMPJ/Calstabin2^-/-^ mice, further confirming the crucial function of Calstabin2 in modulating Ca^2+^ cycling in the intact hearts. This *in vivo* observation is highly consistent with previous landmark studies demonstrating the specific mechanism of RyR2 stabilization. As shown by Wehrens et al., the compound JTV519 effectively increases Calstabin binding to RyR2, thereby reducing SR Ca^2+^ leak and improving cardiac contractile function in heart failure models; critically, this protective effect is completely abolished in Calstabin2^-/-^ mice, underscoring the absolute requirement of the Calstabin2 protein for such pharmacological interventions (Wehrens et al., 2005). Our data utilizing S107 perfectly mirror these fundamental mechanistic principles at the whole-heart level, indicating that without Calstabin2, the pharmacological stabilization of RyR2 cappears difficult to achieve to prevent arrhythmogenic Ca^2+^ events. A seemingly paradoxical finding in our study is the enhanced amplitude of Ca^2+^ transients in Calstabin2^-/-^ mice despite the increased diastolic SR Ca^2+^ leak. While severe SR leak typically depletes Ca^2+^ stores and depresses transient amplitude, the depletion of Calstabin2 sensitizes RyR2 channels to activation. This sensitization dramatically increases the fractional release of SR Ca^2+^ during excitation-contraction coupling. Consistent with previous reports indicating that FKBP12.6 dissociation increases Ca^2+^ transient amplitude (Zhao et al., 2017), this elevated fractional release compensates for the reduced SR load, ultimately generating larger systolic Ca^2+^ transients before irreversible failure occurs. Abnormal dissociation of Calstabin1 from RyRs also contributes to arrhythmia. Because of the embryonic death caused by Calstabin1 knockout (Bellinger et al., 2008), we utilized RC32, a highly specific PROTAC molecule. In our previous studies, this molecule has demonstrated robust systemic efficacy in rapidly achieving Calstabin knockdown across various mammalian models (Sun et al., 2019). Notably, the RC32-treated mice in this study exhibited chaotic Ca^2+^ dynamics that were essentially identical to the arrhythmogenic patterns we observed in the genetic Calstabin2-null models. Although there is a lack of cohort-specific immunoblotting validation, the phenotypic consistency between our pharmacological and genetic models provides strong functional evidence for the critical role of the Calstabin-RyR2 complex in maintaining cardiac Ca^2+^ stability.

It should be noted that optical mapping of a fully beating, intact heart presents challenges regarding spatial resolution. Our study utilized mechanical stabilization, while this approach effectively minimized macroscopic tissue movement, some residual microscopic motion cannot be entirely ruled out. Therefore, the spark-like signals captured in our intact heart preparations are more accurately described as highly localized micro-domain Ca^2+^ events, rather than absolute single-cell recordings.

In conclusion, the present study revealed global chaotic Ca^2+^ events in intact hearts during arrhythmia. These chaotic Ca^2+^ patterns reflect the unsynchronized activity in different regions of the ventricle. Furthermore, because of their superior performance, intracellular spiral waves and ripple waves were successfully visualized during ventricular arrhythmias in intact hearts using the GCaMPJ platform. Different localized regions displayed different patterns of Ca^2+^ waves, indicating that unsynchronized Ca^2+^ events are crucial for initializing and maintaining ventricular arrhythmias. S107 treatment also suppressed chaotic Ca^2+^ events during arrhythmia, indicating that the pharmacological stabilization of RyR2 contributes to the synchronization of intercellular Ca^2+^ activities.

## Data Availability

The original contributions presented in the study are included in the article/**Supplementary Material**. Further inquiries can be directed to the corresponding authors.
