# Supplementary material for: Destabilized calcium dynamics visualized using the genetically-coded probe GCaMPJ in intact hearts of Calstabin2-null mice
Source: Front Physiol. 2026 Apr 29;17:1787427. doi: 10.3389/fphys.2026.1787427 (PMC13167592; doi:10.3389/fphys.2026.1787427)
Supplement: Supplementary file 4 [file SupplementaryFile1.docx]

Supplementary Material

**Supplementary materials for Destabilized Calcium Dynamics Visualized Using the Genetically-coded Probe GCaMPJ in Intact Hearts of Calstabin2-null Mice**

**Figures**

**
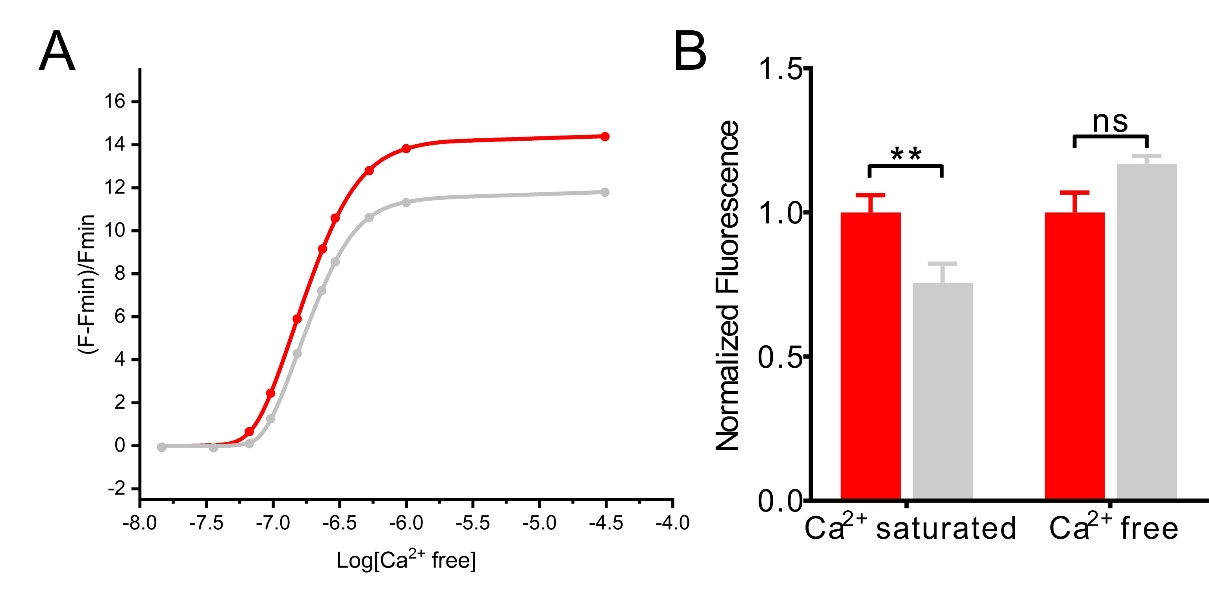
**

**Fig. S1 Ca^2+^ titration assay.** **(A)** The dynamic range of GCaMPJ (Red) and GCaMP6s (gray) in MOPS buffer. **(B)** Normalized fluorescence intensity of Ca^2+^ saturated state (1 mM Ca^2+^) and Ca^2+^ free state (10 mM EGTA) of GCaMPJ monomer (red) and GCaMP6s monomer (gray) in MOPS buffer. Fluorescence intensity was normalized to the brightness of GCaMPJ in both states. All statistical data are represented as mean ± SEM and analyzed using a two-tailed unpaired Student’s t-test. n=8, ^**^*p*＜0.01.


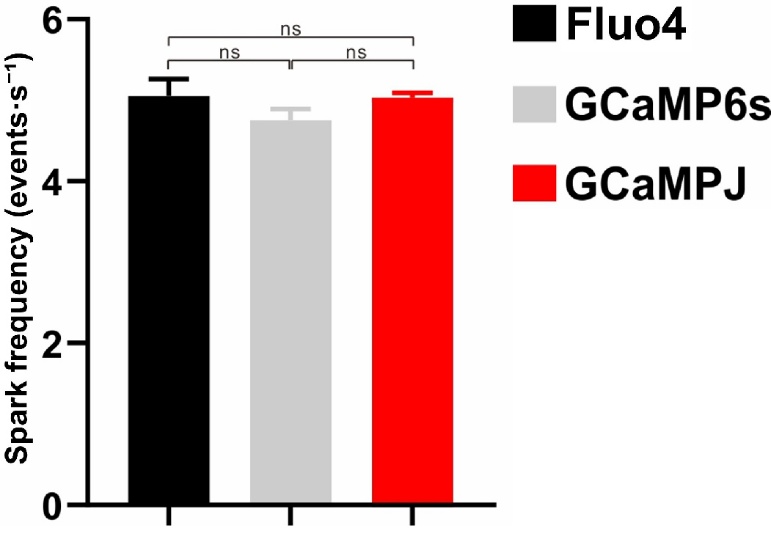


**Fig. S2 The quantification of Ca^2+^ spark frequency.** All statistical data are represented as mean ± SEM and performed two-tail unpaired Student’s *t*-test.


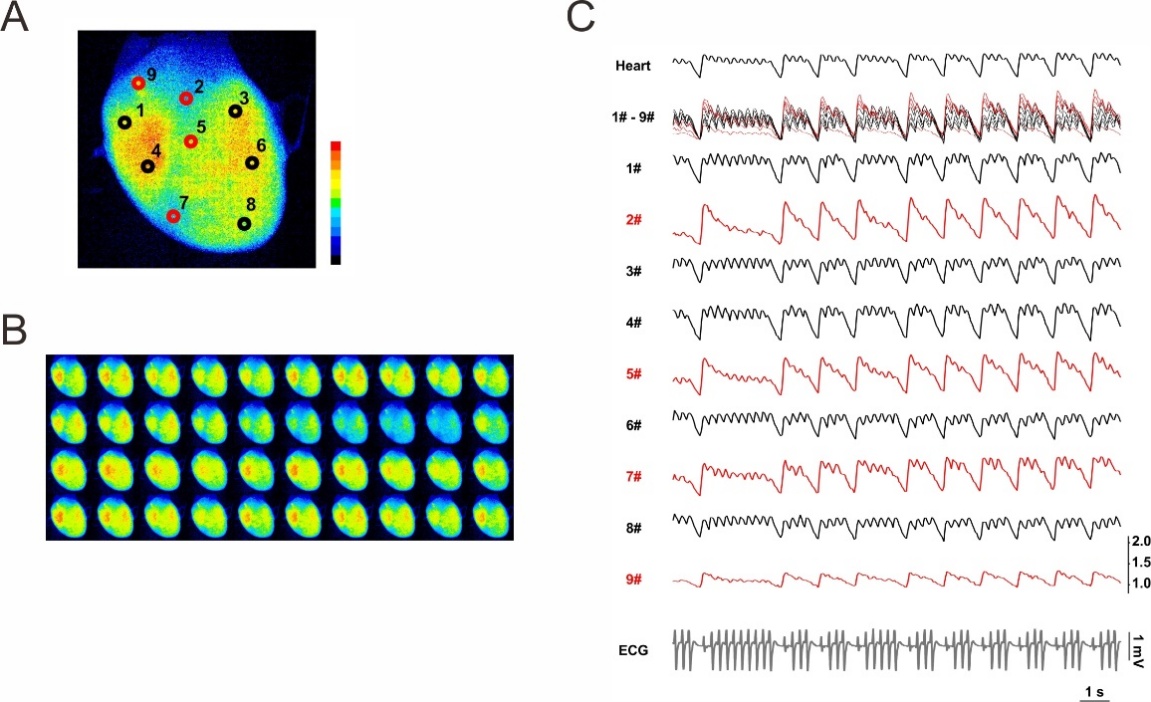


**Fig. S3 The Ca^2+^ changes and ECG during arrhythmia.** **(A)** The pseudo-color map of the heart. Circled numbers represent the random detection areas of the Ca^2+^ signal. **(B)** Representative sequential photographs of the Ca^2+^ activities in intact hearts from αMHC-GCaMPJ mice. **(C)** Nine curves correspond to the fluorescence intensity changes of nine areas described in (A). The bottom trace shows the ECG, which was recorded simultaneously with the Ca^2+^ activities.
